# It Is Not About AI, It’s About Humans. Responsibility Gaps and Medical AI

**DOI:** 10.1007/s11673-025-10423-w

**Published:** 2025-06-26

**Authors:** A. Giubilini

**Affiliations:** Uehiro Oxford Institute, University of Oxford, Littlegate House, 16–17 St Ebbes St, Oxford, OX11PT United Kingdom

**Keywords:** Medical AI, AI ethics, Responsibility in healthcare, Professionalism, Accountability

## Abstract

A lot of the language we use to refer to AI, including in healthcare, uses terminology that originally and literally applies to humans and human relationships. Such terminology includes both non-evaluative terms, like “learning,” “memory,” or “intelligence,” and evaluative terms, like “trust” or “responsibility.” In this article I focus on the latter type and the way it is applied specifically to the case of medical AI. Focusing on the discussion of “responsibility gaps” that, according to some, AI generates, I will suggest that such terminology is revealing of the nature of healthcare professional obligations and responsibility prior to and independently of the assessment of the use of AI tools in healthcare. The point I make is generalizable to AI as used and discussed more broadly: the language used to refer to AI often tells more about humans and human relationships than about AI itself and our relationship with it. In healthcare, whatever else AI will allow us to do, it can prompt us to reflect more thoroughly on professional responsibility and professional obligations.

## Introduction

We have got used to claims about how AI can “revolutionize” healthcare (Savulescu et al. 2024; Vandersluis and Savulescu 2024; Bélisle-Pipon et al. 2021; Mills 2020). The potential of AI for “enhancing operational efficiency and reducing costs, improving diagnostic ability, uncovering new therapeutic targets, and enabling increasingly personalized treatment” (Dzau et al. 2023) is widely acknowledged. Some even refer to a “paradigm shift” (Grote and Berens 2024) in healthcare created by AI or to AI “exceptionalism” to refer to the idea that the introduction of AI might require different ethical frameworks from the standard ones currently adopted (Bélisle-Pipon et al. 2021; Bærøe et al. 2020).

Time will tell whether AI will live up to expectations and whether it will require dramatic changes in the way we conceive of healthcare and healthcare ethics. It is worth emphasizing that very few AI tools have transitioned from research and proof-of-concept models into clinical practice (Lekadir et al. 2023). However, proof-of-concept models have shown a lot of potential

… for lung cancer screening, to diagnose diabetic retinopathy through optical computed tomography scans, diagnosing glaucoma, classifying skin cancer, assessing cardiovascular risk factors, diagnosing a range of paediatric diseases through electronic health records (among many other use cases), and predicting risk of suicide attempts. (Mishra et al. 2023)

While we wait for the revolution to happen, there is a different, less revolutionary type of contribution that AI is already making to healthcare. I want to suggest that the *language* we use to characterize AI’s role can help us better understand the role of human practitioners in healthcare, prior to and independently of the introduction of AI. The terminology we use to describe our relationship with AI often reveals as much about us as about AI’s actual potential for helping us in various tasks. For instance, the terminology of “responsibility gaps” created by AI or that of “trust” in AI can be revealing of what “responsibility” or “trust” means in human interactions more generally, and in the relationships between patients, healthcare professionals, and healthcare systems more specifically. In particular, in this paper I will focus on the way in which the language of responsibility and responsibility gaps in the discussion of AI in healthcare raises questions about the responsibilities of healthcare professionals in the first place. My suggestion will be that—regardless of what AI will be able to contribute to healthcare—a philosophical approach to such technology can provide a better understanding of the role of humans and of their responsibility in the healthcare professional context.

## AI Terminology and Human Terminology: A Revealing or Confusing Relationship?

The contribution of AI to a better understanding of ourselves derives, in part, from the fact that we tend to apply to AI terminology whose meaning originally refers to humans. Floridi and Nobre (2024) have argued that the language of AI has evolved via “conceptual borrowing” (Schmitt [1922] 2005) from the language of human brain and cognitive sciences. Machine “learning,” computer “memory,” computer “vision,” “hallucinating,” indeed “intelligence” itself are all examples of this phenomenon. Conceptual borrowing is not innocent because it can create conceptual confusion with ethical implications, for instance in the form of anthropomorphization of machines or, conversely, the so-called “computerization” and following oversimplification of the human mind (Turkle 2005).^1^

Sometimes it is evaluative concepts such as “responsibility” and “trust” that move from their original domain—that of humans and the ethics of their relationship—to the domain of AI. One result might be not only conceptual confusion, but also, consequently, ethical confusion about what we may or may not expect from AI and what we can hold ourselves responsible for when using it. Some of the ethical and conceptual problems with using human, morally loaded terms to describe AI have been explored in the literature (Deroy 2023). However, we might ask if something positive could derive from this type of conceptual borrowing of morally charged terminology. Here I want to focus on the potential of philosophical reflection on AI terminology for clarifying ethical aspects of human relationships, and particularly professional relationships in healthcare, when human terms are used to refer to it.

For instance, large language models (LLMs) could be used to make initial diagnoses

based on natural language descriptions of patients’ symptoms, health records, and so on […]. To give a fictional example, when a patient has chest pain, a red face, and is sweating, a large language model might infer that she has a high risk of suffering from a heart attack. (Grote and Berens 2024).

This is all good as far as LLMs are, statistically, better than a doctor at diagnosis. However, because AI will make mistakes, questions about trusting AI and the responsibility of AI for mistakes inevitably arise. Whether talk of trust and responsibility regarding AI is even meaningful depends on what trust and responsibility in healthcare delivery are in the first place. AI tools, so to speak, hold up a mirror for us to see what it is exactly that we are doing when we enter into relationships based on responsibility and trust, such as a doctor–patient relationship.

There are two types of processes worth noting. One is linguistic, and it happens when we apply morally loaded terminology to AI as if AI was itself human, that is, we use metaphors. This is something we can see, for instance, in discussion around whether we can trust AI or AI is trustworthy. This discussion provides a good example of the same conceptual confusion that “conceptual borrowing” of non-evaluative terms (“learning,” “memory,” “intelligence,” etc.) risks creating, especially when we forget that we are using metaphorical language, and we treat it as literal. Some have suggested that it would be better to drop the language of trust and trustworthiness to refer to medical AI and speak instead of “reliance.” Unlike trust, reliance does not involve some notion of “goodwill” of the trustee that would be problematic to attribute to an algorithm but is merely about predictability of behaviour (Kerasidou et al. 2022). Elsewhere, I have suggested that by asking ourselves whether we can trust AI, we assume that trust is the kind of things that can be applied to our relationship with AI, and therefore the question whether we can trust AI is really a question about what trust between people is (Giubilini 2024). Similarly, and more generally, it has been argued that robots, by prompting questions about how we differ from them, also prompt us to think about what it is to be human (Turkle et al. 2006; Broadbent 2017).

The second type of process happens when AI is taken not to imitate or replace but to remove ethical dimensions of human relationships. This is the case, for instance, when we ask whether AI can remove substantive aspects of human responsibility without being able to replace it itself, therefore generating a “responsibility gap” (Matthias 2004). When someone relies on AI for certain tasks, such as providing a diagnosis, and accepts that outcome as the valid one without knowing how the outcome was obtained, is the person responsible if the outcome is the wrong one, for example, a wrong cancer diagnosis? In this article, I do not intend to answer this type of questions. Instead, I want to emphasize how asking this type of question can prompt us to clarify what responsibility means within different types of human relationships, including in healthcare.

## Responsibility and Responsibility Gaps

### “Gaps”

There is a debate about whether healthcare professionals using AI for diagnostic purposes retain their responsibility for misdiagnosis, harm to patients, negligence, or any other breach of professional standards (see, e.g., Danaher 2022; Di Nucci 2020; Lang et al. 2023). This is part of a broader problem about potential responsibility gaps that are thought to exist when AI removes or significantly affects some aspect of human decision-making that are considered necessary for responsibility.

Some epistemic and control conditions are typically taken to be necessary for attribution of responsibility (Fischer and Ravizza 1998). Epistemic conditions refer to what an agent should know or reasonably be expected to know to be considered responsible for an outcome. Control conditions refer to the power an agent should have to influence a certain outcome in order to be considered responsible for it. The opacity of many AI applications—that is, the fact that the procedures through which a certain outcome is produced cannot reasonably be expected to be known by users or developers—are sometimes taken to imply that epistemic and control conditions for responsibility cannot be satisfied. For instance, in the healthcare context, AI might produce a cancer diagnosis without the doctor knowing (and therefore being able to explain) how it got to that diagnosis and how any mistake the AI might have made could have been prevented or rectified. According to Andreas Matthias, human inability to meet the epistemic and control conditions necessary for responsibility derives from the fact that “the rules by which [the machines] act are not fixed during the production process, but can be changed during the operation of the machine, *by the machine itself*” (Matthias 2004, 177). Thus, developers cannot take on themselves the responsibility that is not put on doctors. If we cannot reasonably be expected to know those rules, we cannot satisfy epistemic and control conditions. Matthias uses, among others, the example of automatic diagnosis of lung cancer that is constructed so that it minimizes the risk of false negatives but at the cost of increased risk of false positives. Although the latter is not life-threatening and overall this precautionary approach might be justified, false positives can create significant financial and emotional problems as well as side effects of unnecessary therapies for which, it seems, no one is responsible (Matthias 2004, 177). According to Matthias (2004, 177), “nobody has enough control over the machine’s actions to be able to assume the responsibility for them.” Similar conclusions have been reached by authors working in other areas of AI application, such as “killer robots” (Sparrow 2007).

The “gap” in responsibility results from the fact that that machines cannot take on the responsibility they allegedly remove when they are, in some relevant sense, autonomous. Claims that AI health technology is becoming autonomous (Ho 2020; Char et al. 2020 might be true in a specific sense of “decision-making” and “autonomy.” However, it is sui generis decision-making and autonomy that we are talking about. AI tools do not have the kind of agency that is relevant for attribution of responsibility, such as intentions expressing a system of values or the capacity to suffer some kind of punishment for one’s actions, for instance being appropriate target of blame or other appropriate reactive attitudes (Strawson 1962), feeling ashamed, or suffering legal consequences (Sparrow 2007).

In any case, I want to separate here the question of whether artificial agents can be held *responsible* (in either of the senses explained later on) from the question of whether they can be moral *agents*. On some understandings (e.g., Floridi and Sanders 2004; Wallach and Allen 2009), one entity can be the latter without the former. Here I am only concerned with responsibility, regardless of what this discussion implies for the attribution of (some sense of) moral *agency*. Likewise, I will not explore the possibility of “distributed responsibility” in human–machine interactions as discussed for instance by Strasser (2022), because my discussion is limited to cases in which the final decision (e.g., to accept a diagnosis or a treatment recommendation) is up to the human agent. That is, in Strasser’s terminology, I am referring to cases of “tool use” of AI, in which the issue of distributing responsibility that can arise when AI plays more complex social roles (i.e., when it becomes a “social interaction partner”) does not arise. In Strasser’s words, “[r]egarding tool use, a reduced attribution of moral responsibility with respect to the human agent cannot justify assigning the rest of the moral responsibility to the tool. Tools can only be causally responsible” (Strasser 2022, 529). This is precisely what generated the question of whether there are responsibility gaps: do AI tools actually remove substantive aspects of human responsibility, without being able to take them on themselves?

### Responsibility

Now, before even asking whether these AI tools do (in fact) or might (in principle) remove substantive aspects of the type of responsibility that doctors have in their professional relationships with their patients, we need to ask what exactly that responsibility consists of. Santoni de Sio and Mecacci (2021) have argued that discussion about responsibility gaps in AI typically fails to acknowledge the different senses in which we talk about responsibility and the different senses “responsibility” has in the philosophical literature. That is not only a correct diagnosis of a problem but an underestimation of its magnitude. Not only can “responsibility” mean different things (Fischer and Tognazzini 2011), as suggested by Santoni de Sio and Mecacci’s own taxonomy, comprising culpability, moral accountability, public accountability, and active responsibility. In addition, there are alternative taxonomies on offer in the philosophical literature, different conceptualizations of the same terms, and different analyses of the necessary and sufficient conditions for someone to be considered, in some relevant sense, responsible. David Shoemaker (2011), for instance, distinguishes between attributability, accountability, and answerability to refer to different dimensions of responsibility.

On Shoemakers’ taxonomy, an action (or an omission, or an attitude explaining an action) is *attributable* to someone when it reflects the agent’s self in a way that makes the agent suitable for moral appraisal (say, blameworthy or praiseworthy); thus, attributability targets the character of a person. For instance, if I exceed the speed limit because I want to be home on time to watch a football match and, as a result of that, I run over someone, I am blameworthy for the harm I caused, as that expresses my lack of sufficient concern for the safety of others.

On the same taxonomy, someone is *answerable* for an action if there is some rational connection between relevant aspects of one’s self (say, one’s evaluations) and one’s attitude or actions, whereby one is under an obligation or expectation to explain to others why one acted in a certain way. That typically happens when someone’s attitudes or behaviour violate some expectation or norm that is important in a relationship; for instance, a husband not picking up his wife’s hint about the anniversary present she desires, to use Shoemaker’s own example. Thus, answerability targets the deliberation process of an agent, rather than the character of the person.

Attributability and answerability might well overlap, of course. They are both dimensions of *being* responsible, on Shoemaker’s account. But there is a separate dimension, which is about *holding* responsible and which Shoemaker refers to as being “accountable.” *Accountability* is about being required to provide explanations or accepting some form of punishment (blame, disapproval, legal consequences, and so on) for attitudes or behaviours that Shoemaker calls “relationship-defining,” that is, that contravene norms and expectations that are constitutive of a certain relationship. For example, a husband cheating on his wife, to again use one of Shoemakers’ examples. In other words, you are accountable for something that threatens to undermine the nature of the relationship itself—be the relationship romantic, friendly, professional, or of any other nature.

Shoemaker claims that other authors tend to conflate these different senses—for instance, on his view, Angela Smith (2007) conflates answerability and attributability and T.M. Scanlon (2008) conflates answerability and accountability. Here I am not interested so much in whether Shoemaker’s taxonomy is better than alternative ones or whether those rather sophisticated conceptual distinctions track anything too interesting at the empirical level. I will use this taxonomy here because it, being so fine-grained, seems more suitable to illuminate different aspects of professional responsibility in the healthcare context—which, again, might or might not track anything too interesting in the real world. Shoemaker’s taxonomy offers a helpful conceptual map for assessing in what sense we can think that AI could, in principle, generate responsibility gaps in a context governed by specific professional obligations.

As Maximilan Kiener (2022, 2024) suggests, sufficient conditions for answerability—and, on Shoemakers’ terminology, also accountability—are the existence of a causal link between a certain outcome and a certain action and the existence of some obligation to prevent the outcome. If one causes or contributes to causing an outcome that one has an obligation to prevent, one might not be blameworthy for that outcome, but one is still answerable for it. Consider the “lorry driver paradox” famously discussed by Bernard Williams (1981, 28), where a lorry driver runs over a child through no fault of his own. The lorry driver is not morally responsible for the death of the child—he did nothing wrong, didn’t break any rule, and could not reasonably have prevented the death. Yet, it is entirely appropriate for the driver to feel regret, to at least apologize to the family, and to offer explanations about his behaviour during the accident. The paradox lies in the fact that this situation seems to contradict the seemingly obvious principle that one only has to apologize or owes explanation for things that one is responsible for. Kiener’s solution to the paradox lies in separating moral responsibility in the sense of attributability or blameworthiness from answerability and accountability (Kiener 2024). That is, one ought to apologise for things one is answerable or accountable for, not just for the things for which one is blameworthy. In the example, the driver is answerable or accountable in Kiener’s and Shoemaker’s sense of these terms, because he has an obligation not to cause harm to children while driving. However, he is not blameworthy.

To compare, consider another example used by Kiener. Suppose you open a door which hits a vase that was behind it and the vase breaks. You are clearly not morally responsible for breaking the vase. In normal circumstances, one cannot reasonably expect that there is a vase behind a door that one is about to open. Therefore, the breaking of the vase is not *attributable* to you in Shoemaker’s sense, that is, it says nothing about your character or your values or your attitudes towards vases—had you had reasons to think that there was a vase behind the door, you’d probably have opened it more cautiously. However, in this case, unlike in the case of the lorry driver, you are not answerable or accountable, either. No explanation or apology is owed, at least according to Kiener. As he puts it, “[t]he relevant difference is that in the vase case there was no obligation to guard against breaking vases, whereas in the lorry driver case there was indeed an obligation to guard against hitting people” (Kiener 2024, 384).

### Responsibility in Healthcare

In normal, everyday circumstances, the standards determining criteria for blameworthiness or the demands for explanation, justification, apologies, and so on are those of more-or-less widely accepted moral or societal norms (a problematic distinction, whose discussion is beyond the scope of this paper). Within healthcare—or indeed in many other professional settings—responsibility is closely related to specific professional roles regulated by specific professional norms that individuals accept once they enter the profession. For instance, for healthcare, professionals have a duty of care and of pursuing a patient’s best interest—within plausible limits—that individuals outside the profession do not have.

Now, we cannot ask if AI creates responsibility gaps in healthcare without asking what type of responsibility healthcare professionals exactly have, and how it can be diminished, erased, or perhaps increased by AI tools. Healthcare professionals might be held responsible or non-responsible for harms, depending on what understanding of responsibility is adopted among the ones discussed in the previous section (or indeed alternative ones). For instance, they could be morally responsible because a certain action is attributable to them and it results in outcomes for which they are blameworthy, but at the same time not answerable or accountable. Consider, for instance, a doctor that refuses to provide a late abortion to a woman who is not legally eligible for it, because of the doctor’s own moral view on abortion. And suppose that there is no other doctor in the area who is willing to provide an abortion. The woman has to continue an unwanted pregnancy. Depending on what your view on the morality of abortion is, you might think that the doctor is morally responsible in the sense of being blameworthy for a decision that is attributable to him—the decision expresses the doctor’s own view on abortion and is explained by it. More particularly, you might (rightly or wrongly, but not unreasonably) think that a doctor has a moral obligation to do the right thing (in your view, providing an abortion) even when the law demands otherwise. In medical ethics, legal, professional, and moral obligations can come apart (Giubilini et al. 2024). Or conversely, you might think the doctor is morally praiseworthy for having saved a human life. However, the doctor might well not be accountable or answerable, as these dimensions of responsibility depend on other types of standards, such as professional guidelines and legal frameworks. In contexts where abortion is not legally permitted and/or considered acceptable by professional organizations, the doctor is neither answerable nor accountable in Shoemaker’s and in Kiener’s sense. There was never a professional or legal obligation to provide an abortion, and therefore abortion provision was never important or even essential to the relationship the doctor had with the woman in question.

Now consider a case where AI might routinely be involved in the future, such as skin cancer diagnosis (Liu et al. 2020).

A doctor who fails to diagnose an early-stage cancer that, as a consequence, develops into an untreatable one might or might not be blameworthy, professionally negligent, or legally liable for the error. In any case, the doctor is, qua healthcare professional, accountable; that is, she would be required to explain why she failed to promote the best interest of the patient and why the patient suffered significant harm as a result of her conduct. That is because promoting a patient’s best interest and preventing harm to patients are, within limits, professional obligations of healthcare professionals. In fact, they are essential to the professional relationship between a doctor and a patient, so they establish doctors’ accountability on Shoemaker’s account. Professional organizations and the law have their own mechanisms to assess professionals’ conduct and their responsibility for harm caused. For instance, the Bolam test can be used to establish negligence on the basis of whether the doctor *“*has acted in accordance with a practice accepted as proper by a responsible body of medical men skilled in that particular art.” Depending on whether the explanations are considered satisfactory by the relevant “tribunals” according to the relevant criteria of the parties involved—moral standards (whatever they are), patients, professional bodies, courts of law—the doctor could be morally blameworthy, actually blamed, professionally sanctioned, legally liable, or any combination thereof.

But suppose it is an AI tool that makes the same error: an algorithm produces a false negative in a skin cancer diagnosis. Even if AI tools are more reliable than human doctors at diagnosis, false negatives, misdiagnoses, and other kinds of errors will continue to occur, hopefully at lower rates. Now, the AI tool cannot be held responsible in any of those senses, of course. A tool cannot be morally blamed, professionally sanctioned, or legally punished. However, as mentioned earlier, the opacity of the system means that epistemic and control conditions typically taken to be necessary for attribution of responsibility in any of the three senses above are not satisfied by the doctor using the AI tool, or indeed by AI developers.

Now, determining whether there actually is a responsibility gap in such cases, and what kind of responsibility it is, requires us to assess how the different notions of responsibility examined above are relevant in the assessments of healthcare professionals’ “responsibility,” prior to and independently of the introduction of AI technology in healthcare.

Before analysing the issue in more detail, it is important to emphasize that the AI autonomy I am assuming, whatever its meaning, is autonomy in the production of certain outcomes, such as diagnoses or treatment recommendations. It’s not the autonomy in the final decision-making—for instance the decision whether to act upon the autonomous recommendation of the AI. In the types of cases I am discussing, the human always remains in the loop. The issue of responsibility gaps I am concerned with is about whether the human can be held responsible for decisions made on the basis of autonomous, opaque AI input.

We could also think of cases in which the final decision-making is left to the autonomous AI itself, in a completely automatized system where patients don’t interact with doctors when it comes to being provided with a diagnosis or discussing and choosing treatment options. In those cases, there might well be an issue of responsibility gaps at the system level—say, a healthcare system that deploys autonomous AI as a decision-maker. I will not deal with that type of case in this paper, but it is plausible to think that the points I will be making are applicable at the system level, too—though further analysis is required about that. That is, to the extent that a system rather than an individual healthcare provider can sometimes be held responsible for decisions and outcomes, there is a question about potential responsibility gaps in the system introduced by an autonomous AI decision-maker.^2^

### Responsibility “Gaps” in Healthcare and What They Can Teach Us

#### Suppose There Is No Responsibility Gap

Remaining with the case of individual healthcare providers using autonomous AI tools, there is disagreement on whether such responsibility gaps exist. Daniel Tigard (2021), for instance, has argued that there is no responsibility gap generated by the use of AI in healthcare. Partly, that is because those who use AI bear causal responsibility that makes them fit for accountability; if AI is opaque, that is simply a factor that humans should consider when deciding to use it (Köhler et al. 2017). And partly it is because the opacity involved in AI technology is not significantly different from the one already existing in the case of human unassisted decision-making, to the extent that often human decisions are autonomous and opaque (e.g., affected by *implicit* bias) but still fitting targets for answerability and attributability.^3^ This is a point sometimes raised in the discussion on responsibility gap in healthcare. After all, routine medical interventions and clinical decision-making are often opaque to healthcare professionals themselves even when they don’t involve AI (London 2019). Yet, we don’t stop holding doctors blameworthy, answerable, or accountable for their treatments just because they lack epistemic access to their own decision-making process.

For the same reason, some argue that doctors who decide to use and defer diagnoses or decisions to AI can be responsible for any bad outcome occurring (Di Nucci 2020; Tigard 2021). In other words, there is no responsibility gap. On this view, our current ways of attributing moral, professional, or legal responsibility to healthcare professionals already account for opacity. In the same way as doctors are considered blameworthy or are exculpated from responsibility when their clinical decisions result in harm to patients, so they can be responsible or exculpated when harm to patients result from them using AI. That they are exculpated does not mean that they are not responsible in the sense of being answerable or accountable. The exact opposite is true: it means that they are indeed responsible in the sense of being answerable and accountable but that the answers and accounts that they provide to the relevant tribunals—patients and their families, professional organizations, courts of law—are deemed satisfactory. For instance, if AI statistically outperforms humans in making a diagnosis, a doctor could answer the relevant tribunals by pointing out that she used a diagnostic tool that was better than her own diagnostic capacity. That is, she acted in a patient’s best interest.

Thus, if we think that there is no responsibility gap because doctors can be responsible for incorrect diagnoses provided by AI, it means that we think of the responsibility of doctors in their professional capacity in terms of answerability, in Kiener’s sense. To recall, someone is answerable for a certain harm if and only if 1) that person has an obligation to prevent that harm and if 2) that person causes that harm, even if the person is not blameworthy. With regard to this first condition, doctors do have a specifically professional obligation to prevent harm to patients and to pursue their best interest (within plausible limits). With regard to the second condition, the harm occurring to patients as a result of AI’s incorrect diagnosis is caused by the doctor’s decision to use AI for diagnosis. Moreover, pursuing a patient’s best medical interest is, in Shoemaker’s terminology, a relationship-defining aspect of the doctor–patient relationship. This is for Shoemaker a necessary condition for accountability for the harm caused.

We can conclude that AI does not create any gap in responsibility only if the responsibility of doctors is understood as answerability or accountability in these two senses. A doctor owes an explanation, perhaps an apology, for an incorrect diagnosis produced by opaque, autonomous AI, even if the doctor is not responsible in the sense of being blameworthy. The explanation might well be a satisfactory one, so that, for example, no professional or legal sanction is warranted.

What matters, for the purpose of this discussion, is that arguing that AI does not create any responsibility gap because professionals using AI are still responsible for the outcomes implies holding a specific view of the kind of responsibility for harm that healthcare professionals have, prior to and independently of the use of AI. It implies the view that healthcare professionals are answerable and accountable—in Shoemaker’s and Kiener’s sense —for harm they cause even when they are not morally blameworthy for such harm.

#### Suppose There Is a Responsibility Gap

Others argue that there can indeed be a responsibility gap when AI is used in healthcare, but that humans can compensate for that through a process of “responsibilization,” which John Danaher describes as follows:

Responsibilisation is where we confront the tragic choice head-on and accept responsibility for resolving the conflict in a particular way. We do not pass the buck to someone else; we do not bury our heads in the sand. We embrace the fact that life sometimes involves these tragic tradeoffs, and we live with the consequences. We accept that it is our moral responsibility to decide. (Danaher 2022, 25)

Lang and colleagues adopt, in the case of healthcare, a collectivist version of this notion in the form of *shared* answerability (in Kiener’s sense). On their view, responsibilization is shared in the sense that “various key stakeholders ought to collectively responsibilize (viz., assume responsibility for) the gaps” (Lang et al. 2023, 52). In the perspective of “value sensitive” design, for instance, moral values—say, respect for privacy—are embedded into technological devices, including AI, by designers (e.g., Friedman 2004; Nissenbaum 2001; Van den Hoven 2008). In such cases, responsibility is distributed between developers and those using such technologies (in this case, clinicians). When bad outcomes occur as a result of technologies operating on the basis of those values, a responsibility “gap” might be generated not at the level of individual user’s responsibility but at the level of the dynamic system constituted by tool-users and designers (Van den Hoven 2013). These might well be the actors engaging in shared answerability.

For the purpose of this discussion, I am interested not so much in the plausibility of the notion of shared responsibilization to compensate for responsibility gaps, but in the reason why the authors think such responsibility gaps exist in the first place. According to Lang and colleagues, the explanations offered above for why AI does not generate a responsibility gap in healthcare are not satisfactory. They claim that saying that a doctor adopting AI has complied with the best standard of care is question begging because it raises the question whether that standard is adequate and therefore whether the doctor is actually fulfilling her professional duty of care (Lang et al. 2023, 52). In other words, we cannot simply assume that by using opaque AI which is, by hypothesis, statistically better than healthcare professionals at diagnosing, a professional is fulfilling the relevant professional obligations.

But what type of responsibility are we now talking about? And what is it, exactly, that a doctor is responsible for? An AI that, hypothetically, performs better than a human means that a patient has a better chance of receiving a correct diagnosis from AI than from a healthcare professional. If we think that there can still be something for which someone or some entity could be (held) responsible in some sense but no one or nothing who can take on that responsibility—and therefore a responsibility gap—we would need a different account of the basis for responsibility, answerability, and accountability of healthcare professionals. That is, a different moral or normative standard (e.g., societal or professional expectations) for which a doctor could be morally blameworthy and professionally or legally answerable or accountable. For instance, the moral, professional, or legal (or any combination thereof) obligation to guarantee a minimum threshold of expected correct diagnosis. Some might suggest that AI cannot provide that and that a healthcare professional, or a healthcare system, would need to discharge that responsibility in some other way that does not necessarily require AI, but might require more time and resources.

That might well be the case, though it remains to be explained how that could be achieved without relying on opaque AI that performs better than humans. Perhaps there is an obligation to spend more time and resources on diagnostics, for instance with doctors being allocated more time, financial resources, and tools to assess a patient. If so, the locus of that responsibility, answerability, or accountability would be at the level of the healthcare system and policy-making. Be it as it may, seeing a responsibility gap created by AI in this context raises, once again, the question of what responsibility exactly a healthcare professional has, such that deferring to an AI diagnostic tool would remove it.

## Conclusion

My main focus in this paper has been not so much on whether there is a responsibility gap, but on the terminology of “responsibility gaps” itself. Particularly in contexts governed by professional standards and obligations that define the nature of a relationship—such as the relationship between doctor and patient—responsibility is not only about blameworthiness, or “attributability,” to use Shoemaker’s terminology. Indeed, in some contexts it is not about blame at all, if the practice of blaming is not essential to the relationship and if the harm caused is not the result of a failure to meet a certain obligation—for instance a minimum threshold of diagnostic accuracy.

If we think that AI does not create a responsibility gap in healthcare because doctors are still answerable and accountable, it means that the relevant sense of responsibility we have in mind when we talk of responsibility of healthcare professionals is accountability and answerability, quite regardless of moral blameworthiness. All that doctors ought to do to discharge their responsibility—as we conceive of the term—is to provide answers and explanations that refer to the standards of professional ethics, such as patients’ best interest.

If, however, we think that there is a responsibility gap created by AI in healthcare delivery, we are raising a question about exactly what normative standards doctors should be held to, prior to and independently of the introduction of AI in healthcare. Acting in the patient’s best interest might well require doing something more than relying on a technology that, although more reliable than human decision-making alone, might not be reliable enough.

Thus, discussing what kind of responsibility “gap,” if any, AI might create prompts us to ask two types of questions: first, questions about what dimension of responsibility—attributability, answerability, accountability—we have in mind when we attribute healthcare professionals responsibility for some harm, prior to and independently of the introduction of AI in healthcare, and second, questions about what type of professional obligations healthcare professionals have, prior to and independently of the introduction of AI in healthcare.

More generally, when we try to apply terminology referring to eminently human dimensions such as trust and responsibility to algorithms, we might be revealing more about human relationships than about AI and our relationships with it. If AI is going to become increasingly more integrated into healthcare, we might paradoxically get a better understanding of the human aspect of being a healthcare professional. Asking questions about responsibility and AI is a way of asking questions about the nature of human responsibility.

## Footnotes

1 As Floridi and Nobre also note, “The situation generates confusion in those who do not know better and believe that AI is intelligent, in those who know better but have faith that AI will create some super-intelligent systems, and in those who may or may not know better but do not care and exploit the confusion for their purposes and interests, often financial” (Floridi and Nobre 2024, 10).

2 I am grateful to an anonymous reviewer for a comment that prompted me to clarify this important point.

3 Tigard writes: “Granted, again, our efforts might not succeed—some AI systems may well remain ‘black boxes.’ However, fully functional adult human beings also fail to provide adequate reasons for their decisions, say, due to implicit biases and this has not stopped us from demanding answers from them. What is important here is that as a social or political process, one with great moral significance, we hold technological systems answerable, even if doing so requires the aid of human experts. Our investigations might fail to produce adequate reasons for questionable outputs, but this is not to say we cannot appropriately make such demands. Further, whether or not satisfactory answers are received, by engaging in answerability practices, we can be better prepared to instigate additional responsibility practices, such as holding rogue systems and any human associates to account.”
